# Fabrication and Optimization of the Thermo-Sensitive Hydrogel Carboxymethyl Cellulose/Poly(N-isopropylacrylamide-co-acrylic acid) for U(VI) Removal from Aqueous Solution

**DOI:** 10.3390/polym12010151

**Published:** 2020-01-07

**Authors:** Juan Tan, Shuibo Xie, Guohua Wang, Chuck Wah Yu, Taotao Zeng, Pingli Cai, Huayong Huang

**Affiliations:** 1 College of Civil Engineering, University of South China, Hengyang 421001, China; Tanjuan0907@163.com (J.T.); wghcsu@163.com (G.W.); chuck.yu@upc.edu.cn (C.W.Y.); biowater@126.com (T.Z.); 2 Key Discipline Laboratory for National Defence of Biotechnology in Uranium Mining and Hydrometallurgy, University of South China, Hengyang 421001, China; 3 Hunan Provincial Key Laboratory of Pollution Control and Resources Technology, University of South China, Hengyang 421001, China; 430000725392@usc.edu.cn (P.C.); hhy@usc.edu.cn (H.H.)

**Keywords:** orthogonal experiments, U(VI), thermo-sensitive hydrogel, adsorption, wastewater treatment

## Abstract

In this work, the thermo-sensitive materials N-isopropylacrylamide (NIPAM) and acrylic acid (AA) were crosslinked with carboxymethyl cellulose (CMC) (CMC/P (NIPAM-co-AA)) via a free radical polymerization method for the removal of U(VI) from aqueous solution. The L16 (4^5^) orthogonal experiments were designed for the optimization of the synthesis condition. The chemical structures of the crosslinking hydrogel were confirmed by FTIR spectroscopy. The microstructural analyses were conducted though scanning electron microscopy (SEM) to show the pore structure of the hydrogel. The adsorption performance of the CMC/P (NIPAM-co-AA) hydrogel for the uptake of U(VI) from simulated wastewater was also investigated. The adsorption reached equilibrium within 1 h. Under the reaction of pH = 6 and a temperature of 298 K, an initial concentration of U(VI) of 5 mg·L^−1^, and 10 mg of the CMC/P(NIPAM-co-AA) hydrogel, the maximum adsorption capacity was 14.69 mg g^−1^. The kinetics fitted perfectly with the pseudo-second-order model, and the isotherms for the composite hydrogel adsorption of U(VI) was in accordance with the Langmuir model. The chemical modification confirmed that the acylamino group played an important role in uranium adsorption. The desorption and reusability study revealed that the resolution rate was still available at approximately 77.74% after five alternate heating cycles at 20 and 50 °C of adsorption-desorption.

## 1. Introduction

The technology of dissolution and leaching is widely used in uranium mining and metallurgy, and a large amount of low-concentration uranium-containing radioactive wastewater can be generated from these industrial processes, which could pose a serious potential risk to the environment and the health of those involved [1]. Traditional methods such as reverse osmosis, ion exchange, and evaporation have the demerits of complexity, high energy consumption, and cumbersome operation [2,3]. The adsorption method, which possesses the characteristics of a wide source of materials, low cost, high selectivity, fast treatment rate, and large capacity would provide a possibility for the efficient uptake of uranium-bearing wastewater [4]. However, adsorbents are usually difficult to regenerate and have poor reusability. Recently, “smart” hydrogels have attracted much interest in the treatment of wastewater contaminated with heavy metals. These “smart” hydrogels are characterized by their high expansion capacity, biocompatibility, reversibility, and maneuverability compared with traditional methods [5,6]. The intelligent responsiveness of hydrogels means that they are “smart” soft materials with three-dimensional cross-linked network structures that can change volume/shape or phase in sensitive response to environmental stimulation including temperature, pH, magnetic field, electric field, light, and chemical substances [7,8,9,10,11]. The response characteristics of “smart” hydrogels are important parameters for their wide application in the fields of drug control release and separation, heavy metal adsorption, and biomedical treatments [5,12,13].

Poly(N-isopropylacrylamide) (PNIPAM) is the most representative temperature-sensitive polymer. It undergoes an abrupt reversible phase transition near the lower critical solution temperature (LCST), exhibiting a dramatic volume change from the swollen network to the shrunken state by expelling free water [14,15]. Even though the PNIPAM hydrogel shows intelligent thermal sensitivity, which is beneficial for the treatment of wastewater containing heavy metals, it exhibits a low capacity of adsorption for metal ions. The presence of functional groups within polymer networks helps in binding heavy metals via the formation of complex structures [16]. Ju et al. [17] prepared poly(N-isopropylacrylamide-co-benzo-18-crown-6-acrylamide) (P(NIPAM-co-BCAm)) hydrogel by crosslinking the ether group with NIPAM. The P(NIPAM-co-BACm) hydrogel adsorbed Pb^2+^ at a temperature lower than the LCST and desorbed Pb^2+^ at a temperature higher than the LCST. The calic-conjugated thermo-responsive poly(N-isopropylacrylamide-co-tetra(5-hexenyloxy)-p-tert-butylcalix [4]arene) (P (NIPAM-co-HBCalix)) has also been manufactured for capturing Ni^2+^, and its adsorption capacity was maintained above 90% after five cycles of adsorption-desorption [18].

Carboxymethyl cellulose (CMC) is a degradation and biocompatibility biomass material containing a large amount of carboxyl oxygen and lone pair electrons in coordinated bonds that form chelates with metal ions [19,20,21]. Chen et al. [22] produced a bio-based adsorbent consisting of polyacrylamide, polyacrylic acid, and CMC (CMC-PAMA) by the thermal crosslinking of sodium carboxymethylcellulose, polyacrylic acid, and polyacrylamide with good stability and recyclability, whose static adsorption capacities for methylene blue (MB) and Pb(II) were, respectively, 1611.44 mg·g^−1^ and 840.11 mg·g^−1^. Sun et al. [23] applied biomaterials (CMC/gelatin/starch) to stabilize FeS nanoparticles for the removal of Hg(II). The experiments showed that the three biomaterial-stabilized nanoparticles could improve the adsorption efficiency significantly. Among those CMC-based polymers, the interactions of the OH group and metal ions would contribute to improve the stability and insolubility of water, which is conducive to the separation of metal ions from water after adsorption [16].

Our experiments aimed to use N-isopropylacrylamide (NIPAM) as a matrix to prepare a temperature sensitive intelligent hydrogel. During gel preparation, polymerization parameters such as polymerization temperature, the ratio of crosslinker to monomer, and the type of polymer composition could contribute to the desirable properties of thermo-sensitive hydrogels [24]. Even though the polymers of NIPAM, CMC, and acrylic acid (AA) in Na-montmorillonite (MMT) have been fabricated [25], the principal element of preparation of thermo-sensitive hydrogel and the influence mechanism are still unclear. Moreover, temperature-sensitive hydrogels prepared by pre-experiment were fragile after the adsorption of water, which did not meet the reusability demands of uranium removal. Therefore, we have attempted to optimize the synthesis condition for the CMC/P (NIPAM-co-AA) hydrogel through L16 (4^5^) orthogonal experiments and determine the dominant factor in the polymerization. The chemical structure, thermostability, and morphology have been represented by a series of characterizations. The behaviors of composite hydrogel adsorption of U(VI), including adsorption kinetics, isotherms, thermodynamics, and reusability performance, were systematically investigated.

## 2. Materials and Methods

### 2.1. Materials

The main materials for the preparation of thermo-sensitive hydrogels of CMC/P (NIPAM-co-AA) include NIPAM, AA, CMC, N,N-methylene bis acrylamide (BIS), ammonium persulphate (APS), and N,N,N′,N′-tetramethylethylenediamine (TEMED). The sources of these reagents/materials are shown in Table 1. Among them, N-isopropyl acrylamide was recrystallized three times in n-hexane; the rest of the materials were of analytical purity.

### 2.2. Preparation of CMC/P (NIPAM-co-AA) Hydrogels by Orthogonal Experiments

The L16 (4^5^) orthogonal experiments were designed for the optimization of the synthesis condition. Intelligent hydrogels were made by free radical polymerization. Recrystallization NIPAM, AA, BIS (crosslinking agent), and CMC (0.6 g of each) were added in sequence to three flasks containing 10 mL of deionized water under the nitrogen gas condition, which was well mixed using an electric mixer. After half an hour of continuously admitting the nitrogen gas to the reaction flasks, the reaction was initiated in the presence of APS (initiator) and TEMED (catalyst), then the mixture was quickly transferred to a cylindrical sealed tube and placed in a water bath for 24 h at a temperature of 0, 25, 35 and 70 °C to prepare the hydrogel.

After polymerization, the composite hydrogel was washed in deionized water over a 48 h period to remove the unreacted polymer monomer, changing the water every 12 h. Finally, the hydrogel was sliced then vacuum freeze-dried over 24 h. The addition amounts of each polymer monomer are shown in Table 2 and Table 3.

### 2.3. Index Test of Orthogonal Experiments

#### 2.3.1. Swelling Behavior of Composite Hydrogel

The water absorption rate (SR) of the CMC/P-(NIPAM-co-AA) hydrogel was determined by the tea-bag method [26]. Composite hydrogel samples were accurately weighed after drying through vacuum freezing in the freeze dryer (FD5-series, SIM). The mass of the dried hydrogel samples were recorded as *W*_g_. The dry gel was placed in empty tea-bags of 5 cm × 6 cm, which was made of plant fiber. The samples in the tea-bags were then immersed in a buffer solution of pH = 2 for 24 h. The data was recorded to obtain the mass of the hydrogel after absorbing water as *W*_s_. The water adsorption ratio (*SR*) of the composite hydrogel was calculated as follows:

(1) $$SR(\%)=\frac{W_{s}-W_{g}}{W_{g}}.$$

#### 2.3.2. Adsorption Capacity of Uranium

The adsorption capacity of uranium was determined by immersing the 10 mg hydrogel in 30 mg L^−1^ U(VI) solutions at pH = 6 and a temperature of 298 K for 2 h. The adsorption uptake amount of U(VI) by the hydrogel (*q*_e_, mg·g^−1^) and the removal rate (*η*, %) were determined by Equations (2) and (3), respectively, as follow:

(2) $$q_{e}=\frac{\left(C_{0}-C_{t}\right)V}{m}$$

(3) $$\eta =\frac{\left(C_{0}-C_{t}\right)}{C_{0}}\times 100\%$$

where *C*_0_ (mg·L^−1^) and *C*_t_ (mg·L^−1^) represent the initial concentration and equilibrated concentration of U(VI), and m (g) and V (mL) are the mass of the hydrogel and the volume of the solution, respectively.

#### 2.3.3. Study on the Structure and Thermal Stability of Gel

The structure of hybrid hydrogels was confirmed by the Fourier-transform infrared (FTIR) spectra of a Nicolet-460 spectrometer (Nicolet, Madison, WI, USA) from the wavenumber range of 400 to 4000 cm^−1^. The thermal stability of the gels was performed by thermogravimetric analysis (TGA) in the temperature range of 30–600 °C with dried gel on a DSC 200-F3 (Netzsch, Bavaria, Germany).

### 2.4. Batch Sorption Experiments

The evaluation of the adsorption properties of the CMC/P (NIPAM-co-AA) hydrogel for the uptake of U(VI) was carried out. Different concentrations of the U(VI) solution (30 mL of each) were shaken on a shaking table at 150 rpm so that proper reaction could take place with a constant temperature and pH value. The pH value was regulated by HCl and NaOH solutions. The solid-liquid separation was then done by filtration. The uranium concentration in the remaining solution was determined by spectrophotometry (WFJ 2000, Unic, Shanghai, China).

### 2.5. Mechanism Analysis of Adsorption on U(VI)

#### 2.5.1. Characterization

The CMC/P (NIPAM-co-AA) hydrogel, before and after the adsorption of U(VI), was characterized by scanning electron microscopy (SEM), mercury intrusion porosimetry (MIP), and X-ray photoelectron spectroscopy (XPS). The SEM images were obtained by a JSM-7500F SEM instrument (JEOL, Akishima, Japan) after quenching in liquid nitrogen. The pore size of the hydrogel before and after U(VI)-loading were examined by MIP AutoPore IV (Micromeritics, Norcross, GA, USA). XPS spectra were acquired on an Escalab 250Xi (Thermo Fisher Scientific, Waltham, MA, USA).

#### 2.5.2. Chemical Modification of the Hydrogel

In order to examine the functional groups that play a major role in the adsorption of U(VI), the surface of the hydrogel was treated in series with methyl alcohol and concentrated hydrochloric acid to shield the carboxyl group, and then with formaldehyde to mask the hydroxyl group [27,28].

Ten milligrams of hydrogel was mixed with 20 mL of methyl alcohol and 0.2 mL of concentrated hydrochloric acid at 20 °C in a reaction flask and was then shaken on a shaking table for 8 h. Ten milligrams of hydrogel was immersed in 10 mL of formaldehyde under the same condition for 8 h. Ten milligrams of the hydrogel was added into the mixture of 20 mL of formic acid and 10 mL of formaldehyde to shield the amide group. Then, after treatment, the hydrogels were all washed three times with distilled water and were added to 5 mg L^−1^ U(VI) solution for 1 h of adsorption reaction. The remaining U(VI) solution was measured by spectrophotometry (WFJ 2000, Unic, Shanghai, China), as mentioned above.

### 2.6. Desorption and Recycling Experiment

The hydrogel was placed under optimal adsorption conditions to achieve adsorption equilibrium and was washed three times with distilled water. The desorption of U(VI) from the hydrogel was monitored by placing the hydrogel in 0.1 mol L^−1^ of HNO_3_ solution at different temperatures, where it was left for between 10 min and 5 h.

The regeneration ability of the hydrogel was evaluated by conducting a heating cycle of adsorption-desorption. The alternated adsorption-desorption cycles were repeated five times at 20 and 50 °C. The remaining U(VI) concentration in the solution was determined by spectrophotometry.

## 3. Results and Discussion

### 3.1. Results and Range Analysis of Orthogonal Experiments

The results of the 5-variable 4-level orthogonal experiment are shown in Table 4. The water adsorption ratio (*SR*) and U(VI) removal rate (*η*) were the two test indexes. This study used a comprehensive scoring method. The results of the range analysis in this orthogonal experiment are shown in Table 5. From the results, the order of the main influencing factors for preparing hydrogels is: polymerization temperature > the dosage of AA > the dosage of CMC > the amount of cross-linker > the amount of Initiator. The best combination of mechanical properties and uranium adsorption properties was A_2_B_3_C_3_D_2_E_1_, which was 0.1 g of CMC, 0.15 g of AA, 0.06 g of initiator, and 0.03 g of cross-linker at the polymerization temperature of 0 °C.

### 3.2. Characterization of FTIR and TGA for Gel

**Figure 1:**
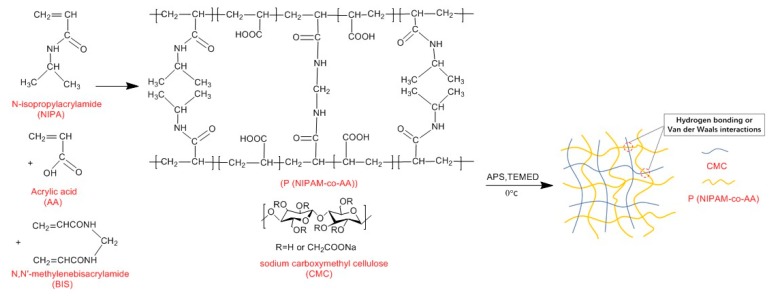
Proposed fabrication process of the CMC/P (NIPAM-co-AA) hydrogel.

**Figure 2:**
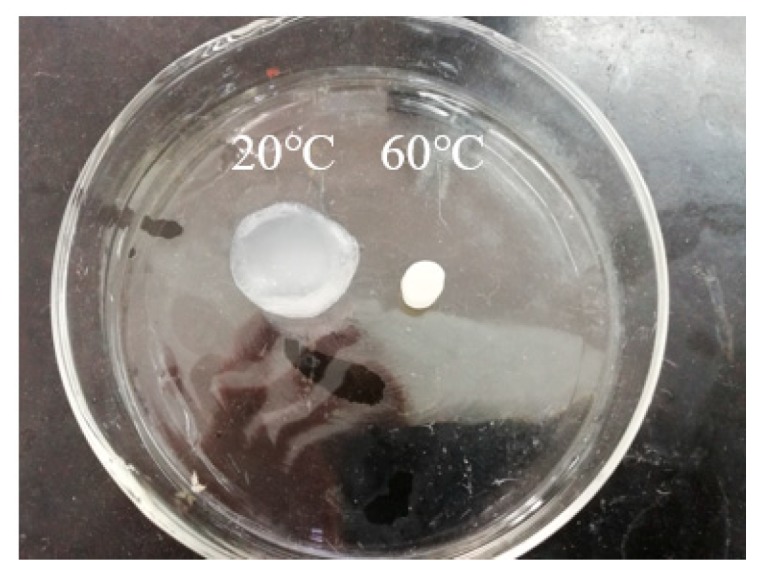
Photographs of the interpenetrating network (IPN) hydrogel after infusion in deionized water at 20 and 60 °C.

Crosslinked network hydrogels are divided into two categories, one is the semi-interpenetrating network (semi-IPN) hydrogel and the other is the interpenetrating network (IPN) hydrogel. The CMC/P (NIPAM-co-AA) hydrogel belongs to the latter. The first layer of the crosslinked network is formed by polymerization, while carboxymethyl cellulose is used as the second layer of the crosslinked network by hydrogen bonding and Van der Waals interactions [29,30]. Figure 1 shows the proposed schematic diagram of the fabrication process of the CMC/P (NIPAM-co-AA) hydrogel [31]. The first network was the formation of the integration of NIPAM and AA, with APS as an initiator; BIS is a cross-linker (a). CMC was immersed in the mixture of the first crosslinked network (b). Finally, the IPN hydrogel was incorporated through the polymerization reaction at 0 °C (c). The photographs of the CMC/P (NIPAM-co-AA) hydrogel after infusion in deionized water at 20 and 60 °C are shown in Figure 2. The IPN hydrogel at 20 °C was translucent in comparison to the hydrogel at 60 °C. There was a very significant discrepancy in the swelling capacity of the gel between 20 and 60 °C. The volume change confirmed the temperature sensitivity of the gel, thus demonstrating the successful synthesis of IPN gels.

**Figure 3:**
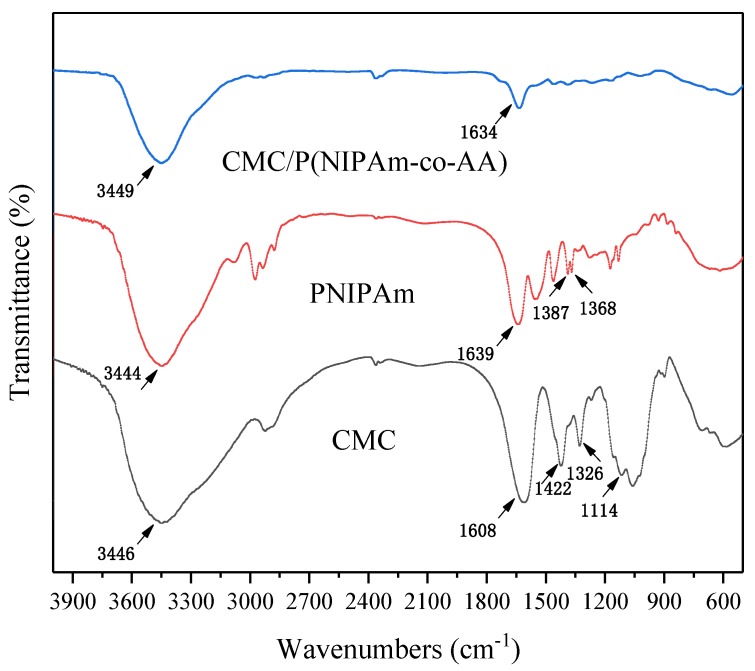
FTIR spectrum of CMC, PNIPAM, and CMC/P (NIPAM-co-AA).

In order to further confirm the IPN crosslinking structure of the gel, the FTIR spectra of CMC, the PNIPAM hydrogel, and CMC/P (NIPAM-co-AA) are presented in Figure 3. (1) In the CMC FTIR spectrum, the absorption peaks at 1608 and 1422 cm^−1^ were symmetric and asymmetric stretching vibrations of the carbonyl group of carboxylic acid, respectively [32]. The adsorption peaks at 1326 and 1114 cm^−1^ belong to the stretching vibration and non-stretching vibration of C–O–C of the cellulose ether bond, while that at 3446 cm^−1^ was from the hydrogen bond with a wide absorption peak [33]. (2) In the FTIR spectrum of the PNIPAM hydrogel, 1639 cm^−1^ indicated the telescopic vibration of C=O of the amide group, 1387 cm^−1^ and 1368 cm^−1^ were the characteristic bimodal of isopropyl of NIPAM, which were the C–H symmetric bending vibration peaks of two methyl groups. The absorption peak of 3444 cm^−1^ was related to the N–H telescopic vibration. (3) In the curve of the CMC/P (NIPAM-co-AA) hydrogel, the absorption peak of 3449 cm^−1^ is much sharper and narrower in comparison to the 3444 cm^−1^ of the PNIPAM spectrum. This is possibly due to the synthesis of the acrylic acid containing O–H group and the covering portion of the N–H peak. At the same time, the peak at 1634 cm^−1^ was the interaction of the bending vibration of C=O and the stretching vibration of N–H. Meanwhile, the peak of the C–O–C of CMC and the isopropyl characteristic peak of PNIPAM basically disappeared in the curve of the CMC/P (NIPAM-co-AA) hydrogel due to the formation of the cross-linking network between the chain of CMC and the polymerase chain of P (NIPAM-co-AA). Therefore, the spectra have illustrated the successful linking of CMC onto the P (NIPAM-co-AA) polymer [34].

**Figure 4:**
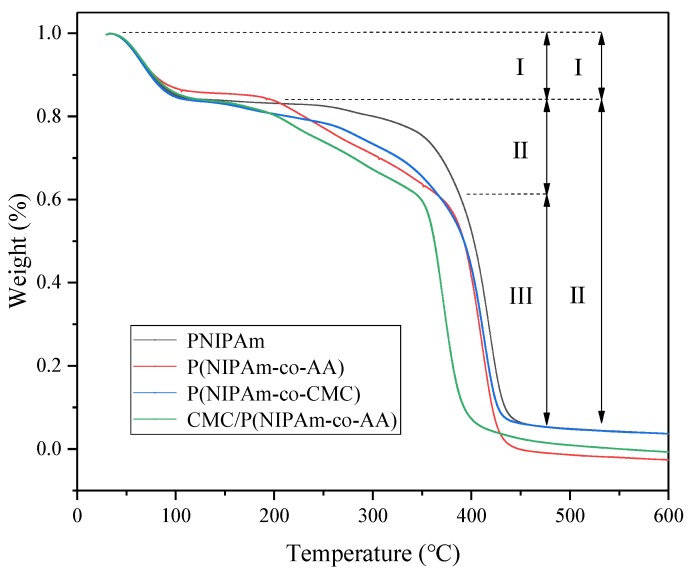
Thermogravimetric (TGA) curves of P-NIPAM, P (NIPAM-co-AA), P (NIPAM-co-CMC), and CMC/P (NIPAM-co-AA) at a heating rate of 10 °C·min^−1^.

The thermogravimetric (TGA) curves of PNIPAM, P (NIPAM-co-AA), P (NIPAM-co-CMC), and CMC/P (NIPAM-co-AA) are presented in Figure 4. The thermal decomposition of P (NIPAM-co-AA), P (NIPAM-co-CMC), and CMC/P (NIPAM-co-AA) is mainly divided into three stages, each with elevated temperatures. The first stage is 50–100 °C and is due to the residual water evaporation. The second stage is 100–370 °C and is mainly due to the fracture and digestion of the terminal and side chain functional groups. Meanwhile, CMC/P (NIPAM-co-AA) was shown to have a large amount of weightlessness in the second stage, indicating that its polymerization chain was fractured and the digestion was more numerous than in the other two materials. The third stage is due to the degradation of the polymerase chain of NIPAM at 370–550 °C. The thermal decomposition of PNIPAM involves only two stages, which are residual water evaporation and self-degradation [35].

### 3.3. Adsorption Performance of U(VI) under Different Conditions

#### 3.3.1. The Contact Time and Adsorption Kinetics Analysis

**Figure 5:**
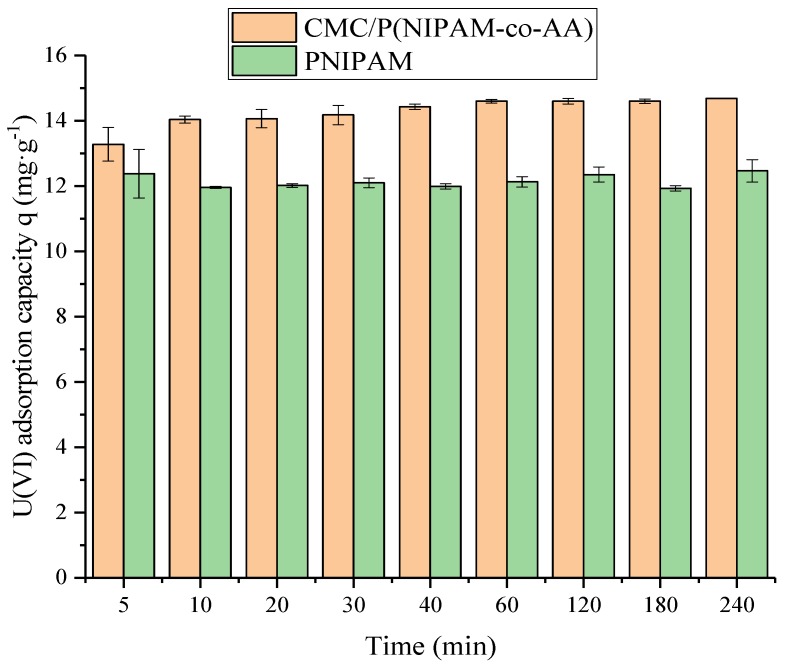
Effect of reaction time on U(VI) adsorption by CMC/P (NIPAM-co-AA) and PNIPAM.

The effect of contact time on the U(VI) adsorption by CMC/P (NIPAM-co-AA) and PNIPAM were evaluated by carrying out an experiment under the reaction of pH = 6 and a temperature of 298 K, an initial concentration of U(VI) of 5 mg L^−1^, and 10 mg of PNIPAM or CMC/P (NIPAM-co-AA), in the range of 5–240 min. The results are shown in Figure 5. The adsorption capacity of the PNIPAM hydrogel for U(VI) was barely immutable over time, which indicates that the adsorption of uranium by the gel is time-independent. For the CMC/P (NIPAM-co-AA) hydrogel, the adsorption reaction reached equilibrium after one hour.

The kinetics of the adsorption of U(VI) by PNIPAM and CMC/P (NIPAM-co-AA) were evaluated by a pseudo-first-order kinetic model, Equation (4), and a pseudo-second-order kinetic model, Equation (5), respectively.

(4) $$\mathrm{In}(q_{e}-q_{t})=\mathrm{In}q_{e}-k_{1}t$$

(5) $$\frac{t}{q_{t}}=\frac{1}{k_{2}\cdot q_{e}^{2}}+\frac{t}{q_{e}}$$

where *q*_e_ (mg g^−1^) and *q*_t_ (mg g^−1^) represent the equilibrium adsorption quantity and the adsorption amount, respectively; *k*_1_ and *k*_2_ are the constant of the first-order adsorption rate and the second-order adsorption rate, respectively; and t (min) is the reaction time.

From Table 6, the correlation coefficient of U(VI) adsorption was matched by the pseudo-second-order kinetic model, *R*^2^, for PNIPAM and CMC/P (NIPAM-co-AA), which were 0.9993 and 1, respectively. Moreover, the theoretical maximum adsorption by the pseudo-second-order kinetic model of CMC/P (NIPAM-co-AA) was 14.71 mg g^−1^, which was much closer to the actual maximum adsorption capacity of 14.69 mg g^−1^. So was PNIPAM, whose theoretical maximum adsorption and actual maximum adsorption capacity were 12.32 and 12.47 mg g^−1^ by the pseudo-second-order kinetic model, respectively. Therefore, the adsorption process of U(VI) by PNIPAM and CMC/P (NIPAM-co-AA) conformed to the secondary dynamic adsorption model, which indicates that the adsorption rate is controlled by chemisorption [36].

#### 3.3.2. Effect of pH

**Figure 6:**
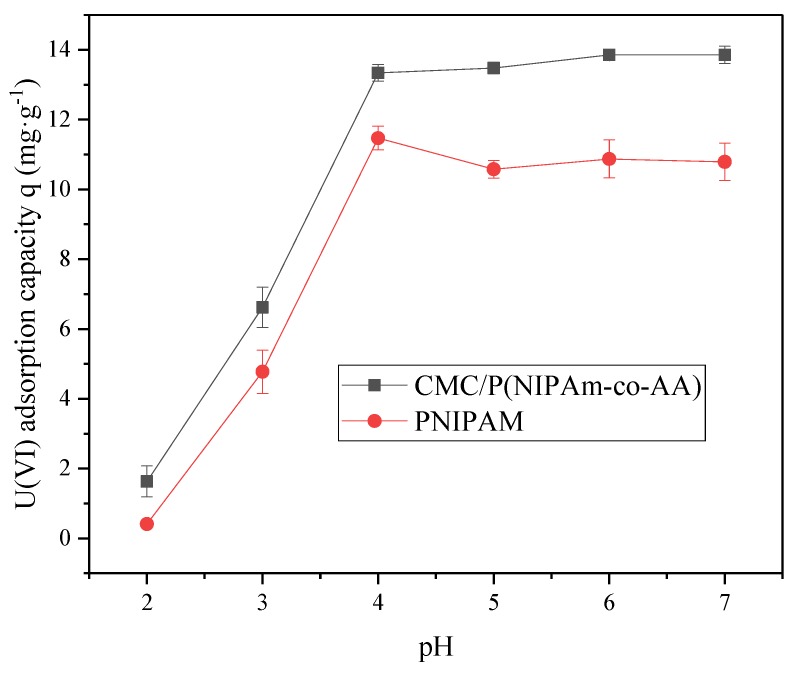
Effect of pH on U(VI) adsorption by CMC/P (NIPAM-co-AA) and PNIPAM.

The effect of pH on the U(VI) adsorption was determined. Ten milligrams of CMC/P (NIPAM-co-AA) or PNIPAM was added to an initial concentration of 5 mg L^−1^ of U(VI), under the reaction condition of 298 K, at various pH values from 2.0 to 7.0 for 1 h of adsorption time. The results in Figure 6 show that the adsorption quantity of U(VI) by CMC/P (NIPAM-co-AA) is higher than by PNIPAM at a pH of 2.0 to 7.0, which illustrates that CMC and AA can promote the adsorption of U(VI) more efficiently. The increase in pH can cause a gradual corresponding increase in the adsorption capacity of CMC/P (NIPAM-co-AA) and PNIPAM. A plateau was reached at pH = 4. At low pH values, the excessive H^+^ in the solution protonates the carboxyl group in the hydrogel, weakening the electrostatic attraction between the carboxyl group and the uranium ion. At the same time, the excess H^+^ generates H_3_O^+^, which competes with the uranium ion for adsorption, thus affecting the U(VI) adsorption of the hydrogel [37].

#### 3.3.3. Adsorption Isotherm

The effect of temperature (293, 303, 308 K) on U(VI) adsorption was studied under the condition of pH = 4. Ten milligrams of CMC/P (NIPAM-co-AA) was placed in the range of 2–100 mg·L^−1^ initial concentrations of U(VI) solutions, over 1 h. The adsorption isotherm was curve fitted by the Langmuir isothermal adsorption model of Equation (6) and the Freundlich isothermal adsorption model of Equation (7).

(6) $$\frac{C_{e}}{q_{e}}=\frac{1}{b\cdot q_{\max}}+\frac{C_{e}}{q_{\max}}$$

(7) $$\mathrm{In}q_{e}=\mathrm{In}k_{F}+\frac{1}{n}\mathrm{In}C_{e}$$

where *C*_e_ (mg L^−1^) is the U(VI) concentration of the adsorption equilibrium, *b* is the constant of Langmuir adsorption equilibrium, *q*_max_ (mg g^−1^) represents the amount of maximum adsorption, k_F_ is the constant of Freundlich adsorption equilibrium, and n is the symbol of adsorption index.

**Figure 7:**
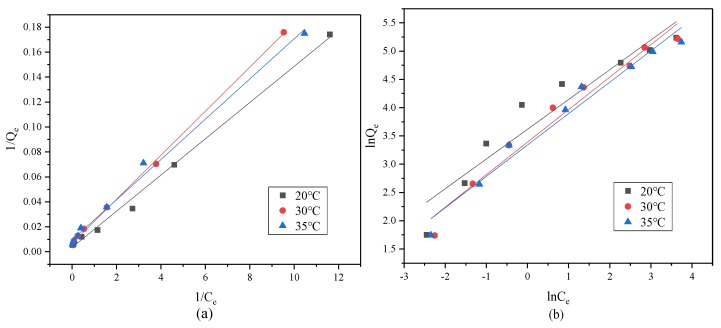
Fitting curve of Langmuir (**a**) and Freundlich (**b**) isothermal adsorption model of U(VI) adsorption by CMC/P-(NIPAM-co-AA).

The adsorption isotherm can be carried out to study the interaction of the adsorbent with the U(VI) to further determine the adsorption mechanism. Figure 7 shows the linear fittings curve of CMC/P (NIPAM-co-AA) corresponding to the models of Langmuir and Freundlich. As the curve exhibits, the adsorption isotherm fits well to the Langmuir isothermal adsorption model. Table 7 is the isothermal adsorption model parameters calculated from fitting curves and equations. From those parameters, the adsorption model of the gel is dominated by the Langmuir model. At lower temperatures, uranium adsorption is more consistent with the Langmuir model, while adsorption is in line with two kinds of isotherm, which reveals that adsorption is more easily carried out on the surface of the hydrogel in a single layer; specifically adsorbed, with the temperature rising, the adsorption mode is uneven. Meanwhile, the adsorption amount is negatively correlated with the characteristic coefficient, b, for the degree of reaction adsorption, indicating that low temperature is favorable for adsorption. The maximum theoretical adsorption capacity is up to 285.71 mg g^−1^. This value is higher than the general hydrogel rather than the smart hydrogel that was determined for the graphene (rGO) hydrogel, with a maximum U(VI) adsorption capacity of 134.23 mg g^−1^ [38].

### 3.4. Characterization Analysis of Hydrogel Before and After U(VI) Adsorption

**Figure 8:**
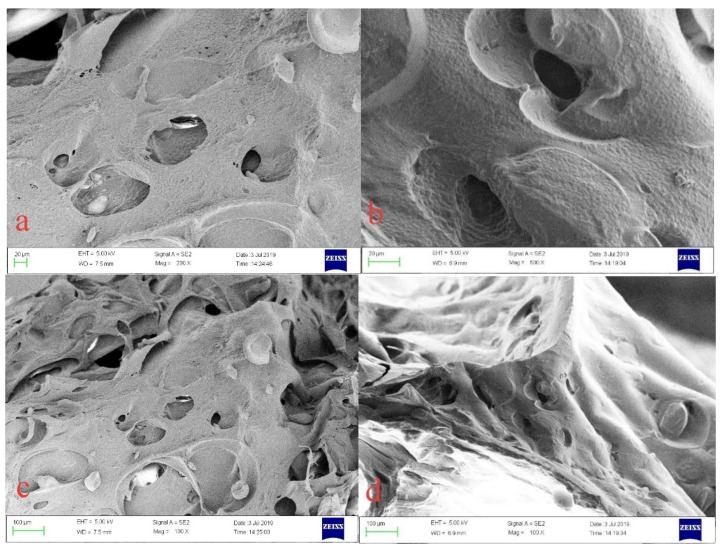
Scanning electron microscopy (SEM) micrographs of the CMC/P (NIPAM-co-AA) hydrogel before and after adsorption of U(VI): (**a**) before adsorption, 20 μm; (**b**) after adsorption, 20 μm; (**c**) before adsorption, 100 μm; and (**d**) after adsorption, 100 μm.

SEM images of the CMC/P (NIPAM-co-AA) hydrogel before (a,c) and after (b,d) adsorption of U(VI) with different magnification are illustrated in Figure 8. The images of Figure 8a,c show the obvious porous structure, but the porous structure of the hydrogel is non-uniform and has irregularities. This is due to uneven polymerization reaction. Moreover, the SEM images after the uptake of UO_2_^2+^ by the adsorbents show a reduced size in porous structure compared with those before the adsorption, which may imply that UO_2_^2+^ has been successfully bound to the adsorption site on the hydrogel. To prove the change in the pores before and after adsorption, a mercury injection test was performed.

**Figure 9:**
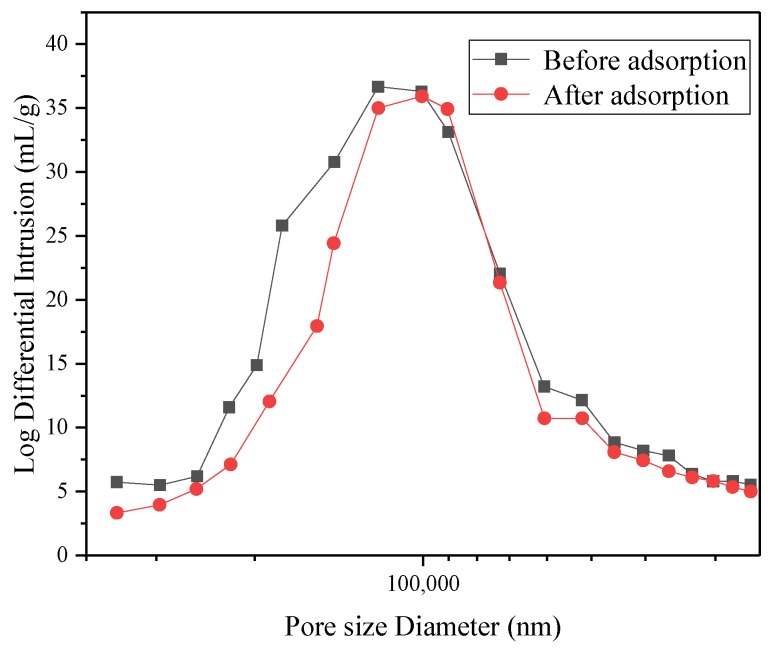
Log differential pore size distribution curves of CMC/P (NIPAM-co-AA) hydrogel before and after U(VI)-loading.

Figure 9 shows the Log differential pore size distribution curves of the CMC/P (NIPAM-co-AA) hydrogel before and after the uptake of U(VI). From Figure 9, pore size diameter is concentrated at 100,000 nm before and after U(VI)-loading, and in Table 8 the average pore diameter before adsorption is much larger than that after adsorption. This result is consistent with previous SEM observations. The phenomenon may result from the fact that the adsorbed U(VI) blocked the channel structure of the hydrogel.

**Figure 10:**
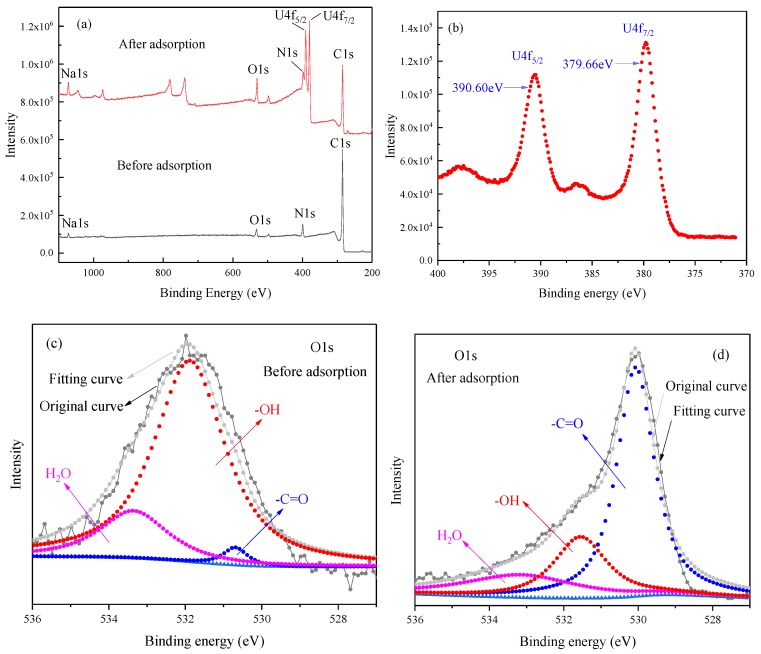
(**a**) XPS spectra of the CMC/P (NIPAM-co-AA) hydrogel before and after U(VI) adsorption, (**b**) high-resolution XPS spectra of U4f, and (**c**,**d**) O1s before and after adsorption.

The XPS spectra of the CMC/P (NIPAM-co-AA) hydrogel before and after the uptake of U(VI) were examined to contrast the possible elemental composition and adsorption mechanism. Comparing the XPS spectra in Figure 10, before and after adsorption, the two sharp and narrow peaks appeared at 391.08 and 379.74 eV, which belong to U4f_5/2_ and U4f_7/2_ [39], respectively. This phenomenon proved that no redox reaction occurred between the hydrogel and U(VI) [40], which further reveals that the uptake of U(VI) on the hydrogel was solely in the form of a positive hexavalent valence state [41]. Three peaks of O1s have been divided, attributing to 530.69 eV for C=O oxygen, 531.88 eV for–C–OH and C–O oxygen, and 533.35 eV for adsorbed H_2_O [42]. The O1s peaks of the carbonyl groups and the hydroxyl groups shifted to low binding energy, which could perhaps be ascribed to the interaction between C=O, –OH, and uranium via the covalent bond [43]. Bonding to UO_2_^2+^ would lead to a decrease of the electro-withdrawing ability of oxygen, so as to increase the electron density on the hydrogel, which in turn reduces its binding energy [44]. In consequence, the dominant mechanism for UO_2_^2+^ sorption on the hydrogel was due to the complexes formed between the oxygen-containing group and U(VI).

### 3.5. Chemical Modification of Hydrogel

**Figure 11:**
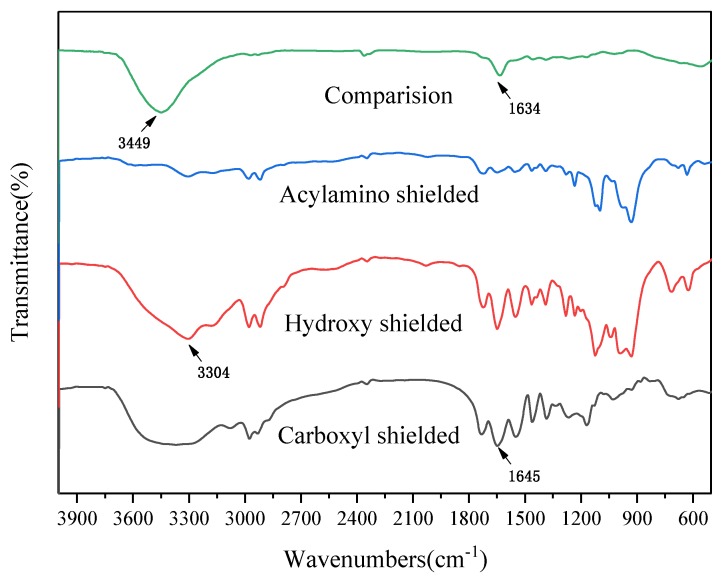
FTIR of CMC/P (NIPAM-co-AA) before and after the functional groups were shielded.

To confirm the successful chemical modification of the hydrogel, the FTIR of CMC/P (NIPAM-co-AA) before and after the functional groups shielded are presented in Figure 11. The curve of comparison is the untreated hydrogel. The peak of 1634 cm^−1^ was changed to 1645 cm^−1^ after the carboxyl groups were shielded, which was attributed to the interaction by the bending vibration of C=O and the stretching vibration of N–H. For comparison, the peak of 3449 cm^−1^ of the hydrogen bond disappeared after the carboxyl groups and the acylamino groups were shielded. After the hydroxyl groups were shielded, the peak of 3449 cm^−1^ of the hydrogen bond turned into 3304 cm^−1^. It has been speculated before that the cross-linking network was formed between the chain of CMC and the polymerase chain of P (NIPAM-co-AA), which caused some peaks to be covered. Interestingly, after chemical modification of the hydrogel, some peaks appeared. This may be attributed to the fact that the chemical modification of hydrogel exposes some parts of the hydrogel.

**Figure 12:**
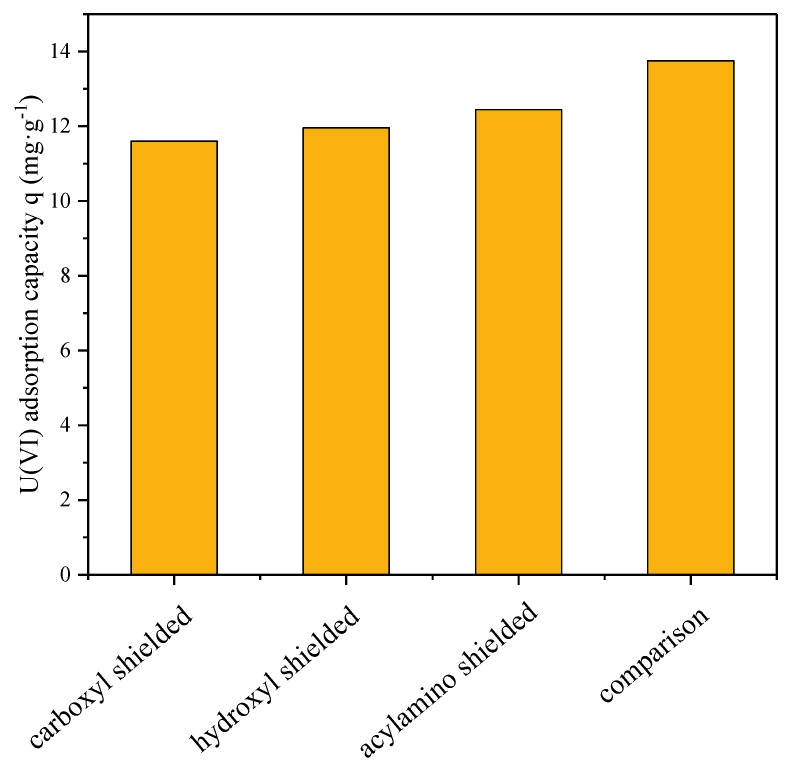
U(VI) adsorption capacity after the functional groups were shielded.

**Figure 13:**
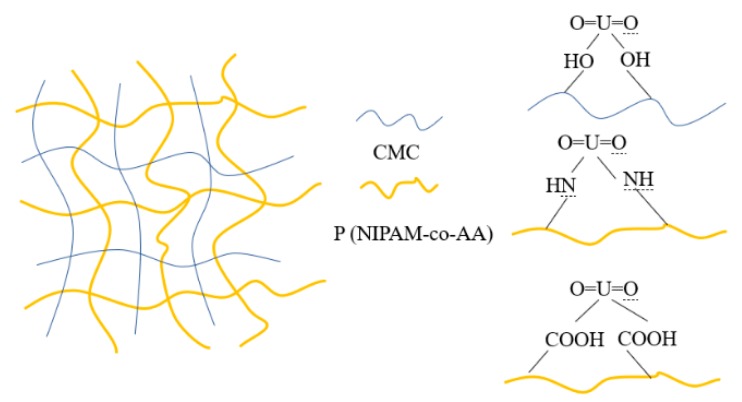
The proposed mechanism of the CMC/P (NIPAM co-AA) hydrogel interaction with UO_2_^2+^.

After the chemical modification of the carboxyl groups, the hydroxyl groups, and the acylamino groups, the gel was added to a 30 mL uranium solution under the optimum adsorption condition of pH = 4 and temperature of 20 °C to analyze the effect of each reactive group on adsorption. From the results in Figure 12, for comparison, the U(VI) adsorption was reduced by 15.58%, 13.01%, and 9.46% after the carboxyl groups, the hydroxyl groups, and the acylamino groups, respectively, were shielded, which indicated that the carboxyl groups, the hydroxyl groups, and the acylamino groups could provide sorption sites for U(VI). Besides, the capability of the gel to adsorb U(VI) was legibly decreased when the –COOH esterifies, while the hydrogel could hardly immobilize U(VI) after –CONH was being shielded. Therefore, the inference was that the acylamino group has a greater impact on the adsorption of U(VI) than the carboxyl groups and the hydroxyl groups, which may be associated with the coordination mode of the functional groups with uranium [45] or more acylamino groups on the surface of the gel [27]. This verdict may provide a reference for the uranium adsorption mechanism. In Figure 13, two oxygen atoms belonging to the carboxyl groups or the hydroxyl groups can bind to uranium atoms. The nitrogen atoms of the acylamino groups can coordinate with UO_2_^2+^ [46,47].

### 3.6. Desorption and Reusability Analysis

To examine the desorption property of the composite hydrogel after adsorption, the gel was adsorbed to equilibrium under optimal adsorption conditions. The sorbed hydrogel was then washed with deionized water and was immersed in 0.01 mol L^−1^ and in 0.1 mol L^−1^ of HNO_3_ solutions separately for different times, the resolution of the U(VI) was calculated, and the result was shown that the hydrogels have a good desorption effect in 0.1 mol L^−1^ HNO_3_ solution, so the 0.1 mol L^−1^ HNO_3_ solution was chosen as the desorption reagent.

**Figure 14:**
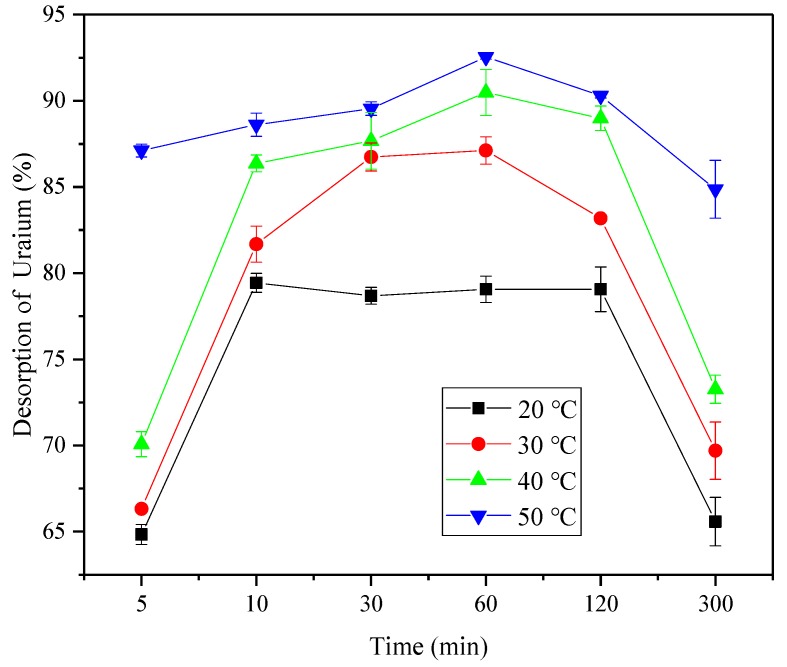
Desorption of Uranium at different temperatures.

As shown in Figure 14, within 5 to 60 min, the rate of desorption of the composite hydrogel increased with time at different temperatures, and the resolution rate increased significantly with the rise in temperature, which verified that the thermo-sensitive hydrogel was “adsorbed at low temperature and desorbed at high temperature”. After desorption for 2 h, the desorption rate decreased at different temperatures, indicating that the hydrogel had different degrees of re-absorption. Among them, the curve decreased most at 20 °C while, at 50 °C, the decline was the slowest, which therefore indicated that the composite hydrogel was suitable for adsorption at low temperatures, and the desorption was faster with a smaller re-absorption amount at high temperatures.

**Figure 15:**
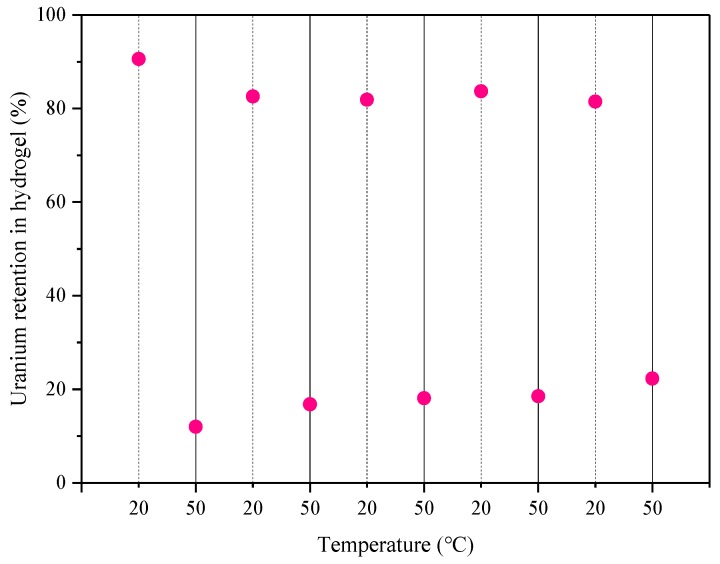
The uranium retention in hydrogel of temperature swing adsorption-desorption.

To examine the reusability of the CMC/P (NIPAM-co-AA) hydrogel, it was subjected to alternate heating at 20 °C and then at 50 °C in five cycles to determine the adsorption-desorption of U(VI) in solution. The five cycles of temperature swing adsorption-desorption are shown in Figure 15. The experiment showed that the resolution rate was still available at approximately 77.74% after five cycles of temperature swing adsorption-desorption. The results have shown that the composite hydrogel has a high desorption efficiency and the sorbed U(VI) can be easily recovered to allow a high recyclability of the hydrogel. Therefore, the hydrogel is suitable for treatment of wastewater containing low concentrations of uranium. However, the specific reason for the amount of loss of the sorbed U(VI) at 20 and 50 °C during the heating cycle is unclear. However, this phenomenon is consistent with Tokuyama’s research [48].

## 4. Conclusions

The optimized preparation method of the thermo-sensitive hydrogel of CMC/P (NIPAM-co-AA) was evaluated by L16 (4^5^) orthogonal experiments. The effect of polymerization temperature on the properties of gels was the most significant of the synthesis factors. The hydrogel was used to determine the U(VI) adsorption for wastewater treatment. The adsorption reached equilibrium within 1 h. Under the reaction of pH = 6 and a temperature of 298 K, the initial concentration of U(VI) 5 mg·L^−1^, and 10 mg of CMC/P (NIPAM-co-AA) hydrogel, the maximum adsorption capacity was 14.69 mg·g^-1^. The kinetics fitted perfectly with the pseudo-second-order model, and the isotherms for composite hydrogel adsorption of U(VI) was in accordance with the Langmuir model. In the desorption and reusability study, low temperature has a beneficial effect for the adsorption of U(VI), while high temperature is good for desorption. The resolution rate was about 77.74% after five cycles of alternate temperature heating at 20 and 50 °C to determine the adsorption-desorption of the hydrogel. The chemical modification of the hydrogel showed that the carboxyl group has the highest adsorption capacity for uranium adsorption. The XPS spectra confirmed that the dominant mechanisms were the complexes between the oxygen-containing group and U(VI) for UO_2_^2+^ sorption by the hydrogel. Therefore, the temperature-sensitive CMC/P (NIPAM-co-AA) hydrogel is a potential intelligent adsorption material for the removal of U(VI) in low-concentration uranium-containing wastewater.

## Figures and Tables

**Table 1:** List of the main materials for the preparation of the temperature sensitive hydrogel.

Materials	Manufacturer
N-isopropylacrylamide (NIPAM)	Chengdu Aikeda Chemical Reagent Co., Ltd. (Chengdu, China)
acrylic acid (AA)	Aladdin Reagent Co., Ltd. (Shanghai, China)
Carboxymethyl cellulose (CMC)	Shanghai shanpu chemical Co., Ltd. (Shanghai, China)
N,N-methylene bis acrylamide (BIS)	Tianjin Kemiou Chemical Reagent Co., Ltd. (Tianjin, China)
Ammonium persulfate (APS)	Xilong Chemical Co., Ltd. (Guangdong, China)
N,N,N′,N′-tetramethylethylenediamine (TEMED)	Tixiai Chemical Industry Development Co., Ltd. (Shanghai, China)

**Table 2:** Level factor of the orthogonal experiment.

Level	A (CMC/g)	B (AA/g)	C (APS/g)	D (BIS/g)	E (Temperature/°C)
1	0.05	0.05	0.02	0.01	0
2	0.1	0.1	0.04	0.03	25
3	0.2	0.15	0.06	0.05	35
4	0.3	0.2	0.08	0.07	70

**Table 3:** Arrangements of the 5-variable 4-level orthogonal experiment.

L_16_(4^5^)	(A) CMC/g	(B) AA/g	(C) Initiator/g	(D) Crosslinker/g	(F) Temperature/°C
1	A_1_0.05	B_1_ 0.05	C_1_ 0.02	D_1_ 0.01	E_1_ 0
2	A_1_0.05	B_2_ 0.1	C_2_ 0.04	D_2_ 0.03	E_2_ 25
3	A_1_ 0.05	B_3_ 0.15	C_3_ 0.06	D_3_ 0.05	E_3_ 35
4	A_1_ 0.05	B_4_ 0.2	C_4_ 0.08	D_4_ 0.07	E_4_ 70
5	A_2_ 0.1	B_1_ 0.05	C_2_ 0.04	D_3_ 0.05	E_4_ 70
6	A_2_ 0.1	B_2_ 0.1	C_1_ 0.02	D_4_ 0.07	E_3_ 35
7	A_2_ 0.1	B_3_ 0.15	C_4_ 0.08	D_1_ 0.01	E_2_ 25
8	A_2_ 0.1	B_4_ 0.2	C_3_ 0.06	D_2_ 0.03	E_1_ 0
9	A_3_ 0.2	B_1_ 0.05	C_3_ 0.06	D_4_ 0.07	E_2_ 25
10	A_3_ 0.2	B_2_ 0.1	C_4_ 0.08	D_3_ 0.05	E_1_ 0
11	A_3_ 0.2	B_3_ 0.15	C_1_ 0.02	D_2_ 0.03	E_4_ 70
12	A_3_0.2	B_4_ 0.2	C_2_ 0.04	D_1_ 0.01	E_3_ 35
13	A_4_ 0.3	B_1_ 0.05	C_4_ 0.08	D_2_ 0.03	E_3_ 35
14	A_4_ 0.3	B_2_ 0.1	C_3_ 0.06	D_1_ 0.01	E_4_ 70
15	A_4_ 0.3	B_3_ 0.15	C_2_ 0.04	D_4_ 0.07	E_1_ 0
16	A_4_ 0.3	B_4_ 0.2	C_1_ 0.02	D_3_ 0.05	E_2_ 25

**Table 4:** Results of the 5-variable 4-level orthogonal experiment.

L_16_ (4^5^)	A	B	C	D	F	Test Index	
						Water Adsorption Ratio (*SR*)	U(VI) Removal Rate (*η*)
1	A_1_	B_1_	C_1_	D_1_	E_1_	41	82.7
2	A_1_	B_2_	C_2_	D_2_	E_2_	13	94.2
3	A_1_	B_3_	C_3_	D_3_	E_3_	37	86.9
4	A_1_	B_4_	C_4_	D_4_	E_4_	3	4.1
5	A_2_	B_1_	C_2_	D_3_	E_4_	22	94.7
6	A_2_	B_2_	C_1_	D_4_	E_3_	25	93.6
7	A_2_	B_3_	C_4_	D_1_	E_2_	21	92.3
8	A_2_	B_4_	C_3_	D_2_	E_1_	50	97.3
9	A_3_	B_1_	C_3_	D_4_	E_2_	12	92.8
10	A_3_	B_2_	C_4_	D_3_	E_1_	18	92.7
11	A_3_	B_3_	C_1_	D_2_	E_4_	28	16.4
12	A_3_	B_4_	C_2_	D_1_	E_3_	4	42.3
13	A_4_	B_1_	C_4_	D_2_	E_3_	37	85.9
14	A_4_	B_2_	C_3_	D_1_	E_4_	25	37.0
15	A_4_	B_3_	C_2_	D_4_	E_1_	43	94.9
16	A_4_	B_4_	C_1_	D_3_	E_2_	12	92.1

**Table 5:** Range analysis of the L16 (4^5^) experimental results.

Analysis	A	B	C	D	E
$\bar{K_{1}}$	24.325	29.150	27.300	23.275	38.975
$\bar{K_{2}}$	30.475	21.000	21.375	32.950	15.375
$\bar{K_{3}}$	16.100	32.925	31.675	23.175	26.425
$\bar{K_{4}}$	30.025	17.850	20.575	21.525	20.150
R	14.375	15.075	11.100	11.425	23.600
Optimal level	**A_2_**	**B_3_**	**C_3_**	**D_2_**	**E_1_**

**Table 6:** Kinetic parameters of adsorption of PNIPAM and CMC/P (NIPAM-co-AA) at 298 K.

Material	*q_e_*,_exp_ (mg·g^−1^)	Pseudo-First-Order Kinetic Model			Pseudo-Second-Order Kinetic Model		
		*k_1_*	*q_e·cal_*	R^2^	*k_2_*	*q_e·cal_*	*R* ^2^
PNIPAM	12.47	0.0124	0.49	0.412	0.1181	12.32	0.9993
CMC/P (NIPAM-co-AA)	14.69	0.0189	0.81	0.848	0.0922	14.71	1

**Table 7:** Langmuir and Freundlich isothermal adsorption model parameters of U(VI) adsorption by CMC/P (NIPAM-co-AA).

T/K	Langmuir			Freundlich		
	*q_max_*/(mg·g^−1^)	*b*	*R*_1_^2^	*k_F_*	*n*	*R*_2_^2^
293	285.71	0.2414	0.9954	36.9439	1.8896	0.9128
303	149.25	0.3807	0.9990	29.6571	1.7391	0.9673
308	105.26	0.5901	0.9937	28.2022	1.8008	0.9690

**Table 8:** Mercury intrusion porosimetry (MIP) parameters of CMC/P (NIPAM-co-AA) hydrogel before and after U(VI)-loading.

Sample	Average Pore Diameter (nm)	Porosity (%)
Before adsorption	90,195.76	97.89
After adsorption	21,469.33	94.82
